# Alcohol significantly lowers the seizure threshold in mice when co-administered with bupropion hydrochloride

**DOI:** 10.1186/1744-859X-7-11

**Published:** 2008-08-18

**Authors:** Peter H Silverstone, Robert Williams, Louis McMahon, Rosanna Fleming, Siobhan Fogarty

**Affiliations:** 1 Clinical Affairs, Biovail Corporation, Mississauga, Ontario, Canada; 2 Research and Development, Biovail Technologies Ltd., Dublin, Ireland; 3 Statistical Group, Biovail Technologies Ltd., Bridgewater, New Jersey, USA

## Abstract

**Background:**

Bupropion HCl is a widely used antidepressant that is known to cause seizures in a dose-dependent manner. Many patients taking antidepressants will consume alcohol, even when advised not to. Previous studies have not shown any interactions between bupropion HCl and alcohol. However, there have been no previous studies examining possible changes in seizure threshold induced by a combination of alcohol and bupropion HCl.

**Methods:**

Experimentally naïve female Swiss albino mice (10 per group) received either single doses of bupropion HCl (ranging from 100 mg/kg to 120 mg/kg) or vehicle (0.9% NaCl) by intraperitoneal (IP) injection in a dose volume of 10 ml/kg, and single-dose ethanol alone (2.5 g/kg), or vehicle, 5 min prior to bupropion dosing. The presence or absence of seizures, the number of seizures, the onset, duration and the intensity of seizures were all recorded for 5 h following the administration of ethanol.

**Results:**

The results show that administration of IP bupropion HCl alone induced seizures in mice in a dose-dependent manner, with the 120 mg/kg dose having the largest effect. The percentage of convulsing mice were 0%, 20%, 30% and 60% in the 0 (vehicle), 100, 110, and 120 mg/kg dose groups, respectively. Pretreatment with ethanol produced a larger bupropion HCl-induced convulsive effect at all the doses (70% each at 100, 110 and 120 mg/kg) and a 10% effect in the ethanol + vehicle only group. The convulsive dose of bupropion HCl required to induce seizures in 50% of mice (CD_50_), was 116.72 mg/kg for bupropion HCl alone (CI: 107.95, 126.20) and 89.40 mg/kg for ethanol/bupropion HCl (CI: 64.92, 123.10).

**Conclusion:**

These results show that in mice alcohol lowers the seizure threshold for bupropion-induced seizures. Clinical implications are firstly that there may be an increased risk of seizures in patients consuming alcohol, and secondly that formulations that can release bupropion more readily in alcohol may present additional risks to patients.

## Introduction

Bupropion HCl is known to cause seizures both when given at therapeutic doses or following accidental or intentional overdose in a dose-dependent manner [1-7]. It is also known that factors which include the excessive use of alcohol and sedatives, history of head trauma or prior seizure, and substance abuse, to mention a few, are associated with increased risk of bupropion-induced seizures [7]. In addition, postmarketing surveillance reports have indicated that there have been rare cases of adverse neuropsychiatric events or reduced alcohol tolerance in patients who are taking alcohol during treatment with bupropion [7]. Despite these latter reports, previous studies of the pharmacokinetic and/or pharmacodynamic interactions between alcohol and bupropion have revealed no significant pharmacodynamic interactions in animals [8], and no pharmacokinetic interactions in healthy human volunteers [9]. Furthermore, there are no studies specifically investigating the interaction between alcohol and bupropion-induced seizures in animals or man. Therefore, the objective of this study was to evaluate the effect of ethanol pretreatment on single-dose bupropion HCl-induced seizures in the Swiss albino mouse model.

## Materials and methods

The study protocol and any amendment(s) or procedures involving the care and use of animals were reviewed and approved by an appropriate ethics committee following internationally approved guidelines (Charles River Laboratories Preclinical Services Inc.'s (CRM) Institutional Animal Care and Use Committee; Charles River Laboratories, Wilmington, MA, USA). During the study, the animals were maintained in a facility fully accredited by the Standards Council of Canada (SCC) and the care and use of the animals was conducted in accordance with the guidelines of the Canadian Council on Animal Care (CCAC).

### Animals

Experimentally naïve female Swiss Crl: CD1 (ICR) albino mice (*Mus Musculus*; Charles River Canada Inc., St. Constant, Quebec, Canada) of approximately 7 weeks of age, and weighing 17.3 to 28.6 g were housed individually in stainless steel wire mesh-bottomed cages equipped with an automatic watering valve in an environmentally controlled vivarium (temperature 22 ± 3°C; relative humidity 50 ± 20%) with a 12-h light/dark cycle. All animals were acclimated to their cages and to the light/dark cycle for 3 days before the initiation of treatment. In addition, all animals had free access *ad libitum *to a standard certified pelleted commercial laboratory diet (PMI Certified Rodent Diet 5002; PMI Nutrition International Inc., St Louis, MO, USA) and tap water except during designated procedures. Animals were randomly assigned to 8 treatment groups of 10 mice per group, using a computer-generated randomisation scheme, ensuring stratification by body weights. Four groups were pretreated with ethanol followed by treatment with increasing doses of bupropion HCl as follows: group 1, ethanol 2.5 g/kg + 0 mg/kg (vehicle); group 2, ethanol 2.5 g/kg + 100 mg/kg; group 3, ethanol 2.5 g/kg + 110 mg/kg; and group 4, ethanol 2.5 g/kg + 120 mg/kg. The other four groups were only treated with the same increasing doses of bupropion HCl as follows: group 5, 0 mg/kg (vehicle only); group 6, 100 mg/kg; group 7, 110 mg/kg; and group 8, 120 mg/kg. The doses of bupropion HCl 100 to 120 mg/kg selected for this study are higher than the low dose of 12.5 mg/kg used in a previous study [8] because more recent studies have revealed that bupropion HCl at low doses of 15 to 30 mg/kg does not induce seizures but protects albino mice against seizures induced by maximal electroshock (anticonvulsant), and at high doses of 100 to 160 mg/kg is proconvulsant in the mice [10]. Animals in poor health or at the extremes of the prespecified body weight range (18 to 30 g) were not assigned to treatment groups and unassigned animals were released from the study.

### Drugs

Bupropion HCl was obtained from Biovail Corporation, Steinbach, Manitoba, Canada, in white powder form. The dose formulations of bupropion HCl were prepared on each day. The appropriate amount of bupropion HCl was weighed and dissolved in an appropriate amount of 0.9% NaCl and then vortexed until a solution was obtained. On each day of treatment, the single doses of bupropion HCl dose were administered by intraperitoneal (IP) injection in a dose volume of 10 ml/kg and dose concentrations of 0, 10, 11, and 12 mg/ml for the 0, 100, 110, and 120 mg/kg doses. The actual dose administered was based on the most recent body weight of each animal. In the applicable treatment groups (groups 1 to 4), each animal was pretreated with ethanol in a dose volume of 10 ml/kg 5 min prior to bupropion dosing. Ethanol was obtained in liquid form from Les Alcools de Commerce Inc., Montreal, Quebec, Canada. Ethanol 2.5 g/kg was administered as a dose volume of 10 ml/kg, and a dose concentration of 0.25 g/ml. Vehicle was 0.9% sodium chloride (NaCl) for injection USP and was obtained from Baxter Healthcare Corporation, Deerfield, IL, USA.

### Study procedure

All animals were examined twice daily for mortality and signs of ill health or reaction to treatment, except on the days of arrival and necropsy when they were examined only once. After the acclimation period and randomisation, on the day prior to the initiation of treatment, all animals were weighed and the individual body weights were used for dose volume calculation. Treatment was then initiated and lasted for 4 consecutive days with equal numbers of animals from each group dosed on each day. On the days of treatment, approximately 5 min prior to bupropion HCl or vehicle dosing, animals in groups 1 to 4 were pretreated with a single dose of ethanol 2.5 g/kg IP in a dose volume of 10 ml/kg. These animals then received the assigned dose of bupropion HCl or vehicle IP. Animals in groups 5 to 8 were not pretreated with ethanol but received their assigned dose of bupropion HCl or vehicle by the IP route. Thereafter, the animals were placed in clear perspex observation boxes containing a foam base for padding and observed for the occurrence of seizures for 5 h, followed by a 5 min assessment at 24 h post dose. The presence or absence of seizures, the number of seizures, the onset, duration and intensity of seizures were all recorded. The intensity of each convulsion was graded using Charles River Laboratories, Inc.'s grading system of mild: head and tail slightly extended and little jerking; moderate: head and tail fully extended and some jerking; or severe: head and tail fully extended and strong jerking. In addition, the presence or absence of ataxic gait, paralysis, and catatonic episodes (without a grading of the intensity or number) were recorded over each 15 min observation period. Any animal that had a single episode of severe seizure lasting longer than 1 min or any animal displaying greater than 40 separate episodes of severe seizures over a 1-h period was sacrificed for humane reasons. At the end of the 5-h observation period, all animals were returned to their home cages, and as deemed necessary, additional bedding, food (on cage floor) and water bottles were provided if an animal was still showing adverse effects from the administration of study drugs.

### Assessment of convulsant activity

The primary outcome variable was the percentage of mice that had seizures. This was the number of animals with seizures (mild, moderate or severe) divided by the total number of animals in each group multiplied by 100. In addition, the convulsive dose of bupropion HCl required to induce seizures in 50% of mice (CD_50_), was calculated for the dose-response curves for bupropion HCl treatment alone and the ethanol/bupropion HCl treatment. The secondary outcome variables were the mean (SD) seizures per mouse in each group, and the duration of seizures.

### Data presentation and statistical analysis

Data was summarised and presented in tables by treatment groups for the primary outcome variable, the percentage of convulsing mice, and the two secondary outcome variables, the mean (SD) seizures per mouse in each group, and the duration of seizures. The CD_50 _values were calculated using the PROBIT procedure in SAS (SAS Inc., Cary, NC, USA). The 95% confidence limits for CD_50 _were calculated according to the method of Litchfield and Wilcoxon [11]. A total of 10 mice per group (total of 40 animals) were used to calculate the CD_50 _for the bupropion alone treatments, and 39 animals for the CD_50 _for the ethanol/bupropion HCl treatments. The number of seizures per mouse was analysed using analysis of variance (ANOVA) on the rank-transformed values, with presence of ethanol (yes/no), bupropion dose, and presence of ethanol-by-bupropion dose interaction as fixed effects in the model. p Values of ≤ 0.05 were considered statistically significant.

## Results

In all groups, except the group treated with vehicle only (group 5), a convulsive effect was observed following the administration of bupropion HCl and/or ethanol. The onset of convulsion was about 9 min following the administration of single doses of bupropion HCl, however, this was highly variable between animals in the same group and across the dose levels for the bupropion HCl alone and ethanol/bupropion HCl treatments. The intensity of the seizures observed following bupropion HCl alone treatment were only mild and moderate (Table 1). Following ethanol pretreatment, overall, there was an increase in the intensity of the bupropion HCl-induced seizures at all the doses. In the 100 mg/kg dose group (group 5), there were marked increases in the number of mild, moderate and severe seizures. In the 110 and 120 mg/kg dose groups, there was a redistribution of the intensity of the seizures resulting in reductions in the mild seizures but a fivefold and twofold increase, respectively, in the moderate seizures (Table 1).

**Table 1:** Effect of ethanol pretreatment on bupropion HCI-induced convulsions: intensity of convulsions

**Dose (mg/kg), n =** ** 10 per group**	**Intensity of convulsions**					
	
	**Mild**		**Moderate**		**Severe**	
	
	**BUP**	**ET + BUP**	**BUP**	**ET + BUP**	**BUP**	**ET + BUP**
0 (V or ET +V)	0	1	0	1	0	0
100	1	37	1	65	0	7
110	21	10	1	5	0	0
120	19	17	2	4	0	0

n = 10 mice per group for bupropion HCl alone and ethanol + bupropion HCl treatment groups.

BUP, bupropion HCl; ET, ethanol; V, vehicle or 0.9% sodium chloride (NaCl).

There were no deaths in the study. One animal treated with ethanol/bupropion HCl 110 mg/kg had excessive convulsions and was therefore euthanised for humane reasons. A variety of clinical signs were observed in the mice following the administration of bupropion HCl, some of which include paralysis, ataxic gait, catatonia, increased respiratory rate, twitching, tremors, increased activity, decreased activity, partially closed eyes, etc. Clinical signs were not dose dependent and pretreatment with ethanol had no effect on the signs observed.

### Percentage of convulsing mice

**Figure 1:**
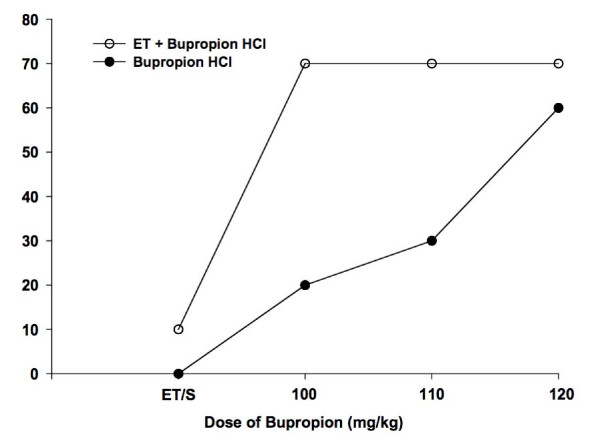
**Dose-response curves of the percentage of convulsing mice following the administration of bupropion HCl alone (closed circles) and the effect of ethanol pretreatment on bupropion HCl-induced seizures (open circles) in the Swiss albino mice**. The 50% convulsing dose (CD_50_) values, the convulsant doses of bupropion HCl required to induce seizures in 50% of mice were 116.72 (CI: 107.95, 126.20) and 89.40 (CI: 64.92, 123.10) mg/kg for the dose-response curves for bupropion alone and ET + bupropion HCl, respectively. Doses of bupropion HCl administered intraperitoneally (IP) were 0 (vehicle or ET + vehicle only), 100, 110, and 120 mg/kg. Ethanol pretreatment was with 2.5 g/kg IP 5 min prior to administration of bupropion HCl. Each data point is the percentage of convulsing mice in n = 10 mice. ET, ethanol + vehicle; S, vehicle (0.9% NaCl).

Administration of single doses of IP bupropion HCl alone induced seizures in mice in a dose-dependent manner with the 120 mg/kg dose showing the largest effect. The percentage of convulsing mice were 0%, 20%, 30% and 60% in the 0 (vehicle only = 0.9% NaCl), 100, 110, and 120 mg/kg dose groups, respectively (Table 2 and Figure 1). Pretreatment with ethanol produced a larger bupropion HCl-induced convulsive effect at all the doses including the ethanol + vehicle only group. There was a marked increase in the percentage of convulsing mice (70% of convulsing mice) at the ethanol/bupropion HCl 100 mg/kg dose, compared to bupropion HCl alone treatment, which was maintained at the ethanol/bupropion HCl 110 and 120 mg/kg doses, resulting in a flat dose-response curve (Table 2 and Figure 1). Ethanol/vehicle (group 1) treatment induced a 10% incidence of seizures.

**Table 2:** Effect of ethanol pretreatment on bupropion HCI-induced convulsions: percentage of convulsing mice

**Dose (mg/kg), n = 10** ** per group**	**No. of convulsing mice**		**Percentage of convulsing mice**	
	
	**Bupropion** ** HCl**	**ET + Bupropion** ** HCl**	**Bupropion** ** HCl**	**ET + Bupropion** ** HCl**
0 (vehicle or ET+vehicle)	0	1	0%	10%
100	2	7	20%	70%
110	3	7	30%	70%
120	6	7	60%	70%

n = 10 mice per group for bupropion HCl alone and ethanol + bupropion HCl treatment groups.

ET, ethanol; vehicle, 0.9% sodium chloride (NaCl).

The CD_50 _or convulsive dose_50_, the convulsive doses of bupropion HCl required to induce seizures in 50% of mice, were 116.72 (CI: 107.95, 126.20) and 89.40 (CI: 64.92, 123.10) mg/kg for the dose-response curves for bupropion alone and ethanol/bupropion HCl treatments, respectively (Figure 1). The CD_50 _of 116.72 (CI: 107.95, 126.20) mg/kg for bupropion HCl alone treatment is similar to the value of 119.7 (CI: 104.1, 137.6) mg/kg reported previously for IP bupropion HCl in Swiss mice [10].

### Mean convulsions per mouse

The analysis of variance results showed a significant overall effect of ethanol pretreatment and bupropion dose on the number of bupropion HCl-induced seizures, and a borderline significant overall ethanol-bupropion interaction effect at the p ≤ 0.10 level (Table 3). Single-dose bupropion HCl alone treatment induced a dose-dependent increase in the mean (SD) seizures per mouse from 0 in the vehicle only-treated group (bupropion HCl 0 mg/kg) to 2.20 (4.49) seizures per mouse in the 110 mg/kg dose group, which was maintained at the 120 mg/kg dose group (mean (SD) convulsions per mouse = 2.10 (1.97)). Pretreatment with ethanol markedly and significantly increased the mean (SD) seizures per mouse compared to bupropion HCl alone treatment only in the 100 mg/kg dose group (ethanol/bupropion HCl = 10.90 (17.28); bupropion HCl alone = 0.20 (0.42); p = 0.0019). There were no statistically significant differences between the mean (SD) seizures per mouse obtained for ethanol/bupropion HCl versus bupropion alone treatments for the 0, 110 and 120 mg/kg dose groups (Table 3).

**Table 3:** Effect of ethanol pretreatment on bupropion HCI-induced convulsions: mean standard deviation (SD) convulsions per mouse

**Dose (mg/kg), n =** ** 10 per group**	**Total no. of convulsions**		**Mean (SD) convulsions per mouse**		
	
	**BUP**	**ET + BUP**	**BUP**	**ET + BUP**	**p Value**
0 (V or ET +V)	0	2	0.00 (0.00)	0.20 (0.63)	0.1027*
100	2	109	0.20 (0.42)	10.90 (7.28)†	
110	22	15	2.20 (4.49)	1.50 (1.72)	
120	21	21	2.10 (1.97)	2.10 (3.35)	

n = 10 mice per group for bupropion HCl alone and ethanol + bupropion HCl treatment groups.

*p Value for overall ethanol-bupropion interaction effect (ethanol effect, overall p = 0.0183; bupropion dose effect, overall p = 0.0007).

†p = 0.0019 for pairwise comparison with corresponding mean value for bupropion alone treatment.

BUP, bupropion HCl; ET, ethanol; SD, standard deviation; V, vehicle or 0.9% sodium chloride (NaCl).

### Duration of convulsions

Administration of single doses of bupropion HCl alone induced only short and medium duration seizures. The number of short seizures increased with dose to a maximum of 22 at the 110 mg/kg dose with a slight decrease to 18 at the 120 mg/kg dose (Table 4). In contrast, pretreatment with ethanol increased the total numbers of bupropion HCl-induced short and medium seizures, as well as caused long seizures. In addition, the number of short, medium and long seizures was markedly highest at the 100 mg/kg dose followed by a marked reduction at the 110 mg/kg dose and a further reduction at the 120 mg/kg dose only for the medium and long seizures (Table 4).

**Table 4:** Effect of ethanol pretreatment on bupropion HCI-induced convulsions: duration of convulsions

**Dose (mg/kg),** **n = 10 per** ** group**	**Duration of convulsions**					
	
	**No. of short convulsions** ** (0 to 10 s)**		**No. of medium** **convulsions** ** (11 to 30 s)**		**No. of long convulsions** ** (≥ 31 s)**	
	
	**BUP**	**ET + BUP**	**BUP**	**ET + BUP**	**BUP**	**ET + BUP**
0 (V or ET +V)	0	1	0	1	0	0
100	1	78	1	17	0	14
110	22	6	0	3	0	6
120	18	17	3	1	0	3

n = 10 mice per group for bupropion HCl alone and ethanol + bupropion HCl treatment groups.

BUP, bupropion HCl; ET, ethanol; V, vehicle or 0.9% sodium chloride (NaCl).

## Discussion

The pharmacokinetic and pharmacodynamic interactions of ethanol with antidepressant drugs are well known [12-17]. Interactions between ethanol and psychotropic drugs could be additive, synergistic (potentiation) or antagonistic [15]. Even though there are published reports of animal [8] and human [9,18] studies investigating the pharmacokinetic and/or pharmacodynamic interactions between alcohol and bupropion, there are no published studies precisely evaluating the effects of alcohol on the convulsive liability of bupropion. This study was therefore designed to investigate the effect of ethanol pretreatment on single-dose bupropion HCl-induced seizures in the Swiss albino mice. The results of the primary outcome variable showed that bupropion HCl alone treatment in the dosage range 0 to 120 mg/kg was associated with a dose-dependent increase in the percentage of mice with bupropion HCl-induced seizures. This finding is consistent with previous reports that indicate bupropion induces seizures in a dose-dependent manner in animals [10,19] and humans [2,3,7]. Pretreatment with ethanol resulted in markedly increased percentage of mice with bupropion HCl-induced seizures at the 100 mg/kg dose, which was maintained at the 110 and 120 mg/kg doses. The latter results are consistent with a 3.5-, 2.3- and 1.2-fold increase in the percentage of convulsing mice at the 100, 110 and 120 mg/kg doses, respectively, following ethanol pretreatment. In addition, ethanol pretreatment resulted in a flat dose-response within the dosage range of 100 to 120 mg/kg studied. The CD_50 _for bupropion HCl alone treatment, a well known index of convulsive liability, of 116.72 (CI: 107.95, 126.20) mg/kg is similar to the value of 119.7 (CI: 104.1, 137.6) mg/kg reported previously for bupropion HCl in Swiss mice [10], and confirms the validity of this animal model. Pretreatment with ethanol resulted in a 23% reduction in the CD_50 _value for bupropion HCl-induced seizures.

The results of the secondary outcome variables were generally consistent with the results of the primary outcome variable. Bupropion HCl alone treatment induced a dose-dependent increase in the mean seizures per mouse up to the 110 mg/kg dose, which was maintained at the 120 mg/kg dose. Ethanol pretreatment resulted in a marked and statistically significant 54-fold increase in bupropion HCl-induced mean seizures per mouse only at the 100 mg/kg dose. There were no significant differences in bupropion HCl-induced mean seizures per mouse at the 110 and 120 mg/kg doses following ethanol pretreatment. With respect to the duration of seizures, bupropion HCl alone treatment only induced short and medium duration seizures, which when combined was dose dependent up to the 110 mg/kg dose. Ethanol pretreatment increased the duration of the seizures overall, resulting in more episodes of short, medium, and long duration bupropion HCl-induced seizures, but particularly in the 100 mg/kg dose group.

The results of this study are in conflict with the results of previous studies that reported no pharmacodynamic interactions between alcohol and bupropion in mice [8], and no pharmacokinetic interactions in normal healthy volunteers [9]. The reason for the discrepant previous results may be because those studies of the pharmacokinetic and pharmacodynamic interactions between alcohol and bupropion in normal healthy volunteers [9,18] used a low dose of bupropion (100 mg, approximately 1.5 mg/kg) that is unlikely to be associated with the occurrence of seizures since bupropion-induced seizures are dose dependent. Similarly, a previous study [8] investigating the interactive effect of combined treatment with alcohol and bupropion in adult albino mice utilised a low dose of bupropion (12.5 mg/kg IP) which is much lower than the convulsive doses of 100 to 160 mg/kg IP, with a CD_50 _of 119.7 (CI: 104.1, 137.6) mg/kg and CD_97 _of 156.7 mg/kg, that were subsequently reported for bupropion in mice by other investigators [10]. In addition, lower doses of bupropion (15 to 30 and 5 to 10 mg/kg, respectively), which did not induce seizures, have been reported to protect against seizures evoked by maximal electroshock [10] and nicotine [20] in mice. However, one group has reported that the combination of bupropion with alcohol abolished the impairment in auditory vigilance and mental slowness observed following the administration of alcohol alone in normal healthy volunteers (a pharmacodynamic interaction) even though they used a low dose of bupropion (100 mg) and found no pharmacokinetic interaction [18].

The mechanism of bupropion HCl-induced seizures is unknown [21,22]. Similarly, the mechanism for the synergistic interaction reported here between ethanol and bupropion HCl is also unknown. This interaction is unlikely to be solely due to pharmacokinetic reasons since a previous crossover study that investigated the interactions between alcohol and bupropion found no such interactions [9]. This previous study, also in normal healthy human volunteers, examined the effect of administration of oral bupropion HCl 100 mg followed by the administration of ethanol found no changes in the pharmacokinetics of bupropion, and vice versa [9].

The observed interaction between ethanol and bupropion reported in the present study has potential clinical implications. It has been recognised that the seizure risk of bupropion is increased in subjects undergoing abrupt withdrawal from alcohol [3,4], hence, bupropion administration is contraindicated in such patients [7]. However, the more recent although rare postmarketing reports of adverse neuropsychiatric events or reduced alcohol tolerance in patients who are drinking alcohol during treatment with bupropion [7], suggests that there is an interaction between alcohol and bupropion following coadministration, consistent with the findings of this study. Consequently, patients should be cautioned to not consume alcohol with bupropion. Nonetheless, there is good evidence that many patients on bupropion, as well as other anti-depressants, continue to use alcohol [23].

In conclusion, the results of this study demonstrate that ethanol pretreatment followed by single-dose IP bupropion HCl resulted in an increase in the number and percentage of convulsing mice, mean seizures per mouse, the intensity, and the duration of the seizures. Following ethanol pretreatment, the CD_50 _for bupropion HCl alone treatment was reduced from 116.7 to 89.0 mg/kg, representing a 23% reduction. The dose-related increase in the percentage of convulsing mice and mean seizures per mouse is consistent with previous reports that bupropion-induced seizures are dose dependent in animals and humans. The observed pharmacodynamic interaction between ethanol and bupropion-induced seizures in this study is novel and the mechanism is unknown. However, it has potential clinical implications for the prescribing of bupropion. It also implies that caution should be used when bupropion is prescribed to patients either using alcohol or at high risk of doing so.

## Competing interests

The authors declare that they have no competing interests.

## Authors' contributions

The study was conceived by PHS and RW, was designed by LM and SF who were also involved in data acquisition, the first draft of the paper was by PHS, it was carried out in part by LM, and the statistical analysis was by RF. Funding for the conduct of this study and the manuscript preparation was provided by Biovail Laboratories International SRL.
